# Identification of diagnostic and prognostic biomarkers for tuberculosis based on plasma proteomics

**DOI:** 10.1371/journal.pone.0339558

**Published:** 2025-12-22

**Authors:** Yan Hu, Chao Quan, Yuanyuan Zhou, Shangyan Liang, Xuan Wang, Jun Li, Wenqi Liu, Yuzhong Xu, Peng Liu

**Affiliations:** 1 Central Laboratory, Wuhan Pulmonary Hospital, Wuhan Institute for Tuberculosis Control, The Hubei Branch of the National Clinical Research Center for Infectious Disease, Wuhan, Hubei, China; 2 Department of Hematology, Zhongnan Hospital of Wuhan University, Wuhan, Hubei, China; 3 Laboratory Department, Shenzhen Baoan District People’s Hospital, Shenzhen, Guangdong, China; 4 National Clinical Research Center for Infectious Diseases, Shenzhen Third People’s Hospital, Southern University of Science and Technology, Shenzhen, Guangdong, China; UMass Chan Medical School Department of Medicine: University of Massachusetts Chan Medical School Department of Medicine, UNITED STATES OF AMERICA

## Abstract

**Background:**

The differential diagnosis between Tuberculosis (TB) and Non-tuberculous Mycobacteria (NTM) has historically been constrained by the inadequate sensitivity and specificity of current diagnostic methods. Furthermore, distinguishing between Active Tuberculosis (ATB) and Latent Tuberculosis Infection (LTBI) poses significant challenges. This study aims to develop a molecular differentiation system for ATB, LTBI, and NTM by integrating plasma proteomics with multi-dimensional analytical techniques, while also exploring key biomarkers associated with disease progression and treatment response.

**Methods:**

Using label-free quantitative technology, we conducted a plasma proteomics analysis across five groups: ATB, LTBI, NTM, Cured Patients (CPs), and Healthy Donors (HD). Differentially Expressed Proteins (DEPs) were identified through screening (FC > 1.5 or <0.67, *P* < 0.05), followed by Gene Ontology/KEGG pathway enrichment, STRING interaction network, and Mfuzz dynamic clustering analysis to systematically elucidate molecular characteristics. Experimental data were validated through a multidimensional quality control system (Pearson correlation coefficient, peptide distribution, molecular weight distribution, etc.). Enzyme-linked immunosorbent assay (ELISA) was employed to detect the plasma expression levels of target proteins across the groups and to facilitate comparisons.

**Results:**

This study identified 1,338 non-redundant proteins across five cohorts. Comparative analysis revealed 142 DEPs across the three comparative groups (ATB, LTBI, and NTM), which were primarily localized in the extracellular domain. Key findings include: 27 DEPs in the ATB-LTBI group, primarily enriched in inflammatory responses (such as A2M, IL-1R2) and epithelial barrier functions (TGM3, KRT3); 69 DEPs in the ATB-NTM group, characterized by significant changes in immunoglobulin light chains (IGLV2–11) and innate immune effector molecules (S100A8); 46 DEPs in the NTM-LTBI group, closely related to lipid metabolism (APOC3) and extracellular matrix remodeling (FN1). KEGG pathway analysis revealed that DEPs in the ATB-LTBI group were enriched in nitrogen metabolism pathways, those in the ATB-NTM group were associated with thyroid hormone synthesis, and the NTM-LTBI group was involved in phagosome function. Dynamic clustering results showed six treatment response modules: Cluster 1/2 (riboflavin metabolism, complement coagulation pathway) were activated post-treatment, Cluster 3/4 (proteasome, cardiac signaling pathway) exhibited partial reversal in expression, and Cluster 5/6 (platelet activation, cytoskeleton) showed delayed regression. Research confirmed 10 differential proteins between the ATB-CPs and ATB-HD groups, including S100A8, LTA4H, and DEFA1B, which constitute a molecular fingerprint specific to ATB. ELISA validation confirmed significantly elevated S100A8 and GPX3 in ATB group, while NTM group showed higher FGB and lower ATRN levels.

**Conclusions:**

This study systematically reveals the plasma proteomic characteristics under infection statuses caused by different mycobacteria. A discrimination framework for ATB/LTBI/NTM was constructed based on disease-specific differential proteins, overcoming the limitations of traditional diagnostic techniques in distinguishing infection states. Through dynamic analysis of six temporal therapeutic modules, the reprogramming patterns of the host protein network during tuberculosis treatment were elucidated. This research lays a multidimensional molecular foundation for the precise typing, personalized treatment, and prognostic evaluation of mycobacterial infections.

## Introduction

Tuberculosis (TB) is a chronic infectious disease caused by infection with *Mycobacterium tuberculosis* (*Mtb*) and is transmitted through the air. It is one of the top ten causes of death globally. According to the 2024 Global Tuberculosis Report released by the World Health Organization (WHO) [1], there were approximately 10.8 million new cases of tuberculosis worldwide in 2023, with a death toll of 1.25 million, making it one of the deadliest diseases caused by a single infectious agent. Moreover, as a high-burden country for tuberculosis, the incidence rate in China is as high as 134 per 100,000, indicating a severe prevention and control situation. Although significant progress has been made in global tuberculosis prevention and control, the enormous base of latent infected populations and the increasing prominence of Nontuberculous Mycobacteria (NTM) infections [2] continue to pose serious challenges to the control of this disease.

The Bacillus Calmette-Guérin (BCG) vaccine is currently the only approved vaccine for tuberculosis; however, its protective efficacy has significant limitations. Studies have shown that the BCG vaccine can achieve a preventive effect of 71%−92% against severe tuberculosis in children, such as tuberculous meningitis and miliary tuberculosis, but its protective effect against adult pulmonary tuberculosis is weaker (approximately 50%) [3,4]. Furthermore, about 90% of individuals infected with *Mtb* enter a latent infection state (Latent Tuberculosis Infection, LTBI), which may progress to active tuberculosis (Active Tuberculosis, ATB) only when immune function is compromised (e.g., during HIV infection, diabetes, or immunosuppressive therapy) [5]. In recent years, the incidence of NTM infections has significantly increased, especially in low-burden tuberculosis countries, where its prevalence has surpassed that of *Mtb* [6]. NTM are widely present in water, soil, and medical equipment [7], and can cause pulmonary symptoms similar to those of tuberculosis; however, their diagnostic and treatment strategies differ from those of tuberculosis, and misdiagnosis may lead to treatment delays and high mortality rates [8].

Currently, the commonly used diagnostic methods for tuberculosis in clinical practice include sputum smear acid-fast staining, culture of *Mtb*, interferon-gamma release assay (IGRA), and molecular detection techniques such as Xpert MTB/RIF [9]. Although sputum culture is considered the “gold standard,” it takes as long as 4–8 weeks and has a positivity rate of only 30%−60% [10]. The sensitivity of acid-fast staining is low (approximately 40%), making it difficult to distinguish between *Mtb* and non-tuberculous mycobacteria. While IGRA can detect tuberculosis infection, it cannot differentiate between ATB and LTBI, and may show cross-reactivity in areas with a high prevalence of NTM [11]. The Xpert MTB/RIF technology can shorten the diagnostic time to 2 hours by detecting mutations in the rifampicin resistance gene, but it has limited ability to differentiate NTM and insufficient sensitivity for extrapulmonary tuberculosis [12]. Furthermore, the effectiveness of existing methods for diagnosing early infections and immunocompromised patients is significantly reduced, resulting in approximately 30% of tuberculosis cases worldwide not being diagnosed in a timely manner. Therefore, there is an urgent need to further identify molecular markers that can serve as diagnostic and prognostic indicators for tuberculosis.

In this study, we constructed a comprehensive immune response profile for populations with ATB, LTBI, NTM, cured tuberculosis (CPs) and healthy controls (HD) by label-free technologies. Through bioinformatics analysis, we identified differential biomarkers capable of distinguishing between *Mtb* infection and nontuberculous mycobacterial infection, as well as key proteins that can differentiate ATB from CPs. This research not only provides a molecular basis for the precise classification of tuberculosis but also lays a theoretical foundation for the development of novel diagnostic reagents and targeted therapies.

## Materials & methods

### Ethical statement

This study was conducted at Shenzhen Third People’s Hospital between September 2023 and June 2024. All study procedures were performed in strict compliance with the hospital ethics committee’s approved guidelines (Approval No. 2023-011-02). All participants gave written informed consent before participating in any part of the study. The research strictly adhered to the ethical guidelines of the Declaration of Helsinki, ensuring the privacy and data security of the participants.

### Study subjects

In the preliminary screening phase, a total of 5 groups of subjects were included (3 cases in each group), and in the subsequent validation phase, 80 subjects were included (16 cases in each group). The baseline characteristics (age and gender) for all participants in both the preliminary screening and validation cohorts are provided in S1 Table to ensure comprehensive demographic transparency. The grouping criteria are as follows: ATB group was diagnosed based on the “Guidelines for the Diagnosis and Treatment of Pulmonary Tuberculosis” by the Chinese Medical Association’s Tuberculosis Branch, requiring abnormal chest X-ray findings, positive sputum smear/culture, a positive interferon-gamma release assay (IGRA), and the presence of typical tuberculosis symptoms; LTBI group consisted of asymptomatic individuals with normal chest X-ray findings, no history of tuberculosis or treatment, and positive IGRA results; CPs group included individuals who completed at least 6 months of standard anti-tuberculosis treatment, had negative cultures, and showed no signs of recurrence; HD group comprised individuals with no clinical symptoms, negative IGRA results, and no abnormalities on chest imaging; NTM group was diagnosed based on a comprehensive assessment of medical history (environmental exposure, surgical/trauma history), clinical manifestations (skin lesions or non-specific symptoms), histopathology (infectious granulomas), and laboratory tests (culture or molecular biological identification as NTM). Additionally, all HIV-infected individuals and subjects receiving immunosuppressive drug treatment were excluded from the study.

### Plasma sample preparation

5 ml of whole blood was collected using heparin anticoagulant vacuum blood collection tubes and centrifuged at 3000 rpm for 10 minutes at 4°C to separate the plasma. The plasma was aliquoted and immediately frozen at −80°C. All operations were completed within 4 hours post-collection to avoid repeated freeze-thaw cycles and to maintain sample stability.

### ELISA detection

Venous blood was collected from participants and plasma was separated according to the method described in section 2.3. The protein levels of S100A8 (Elabscience, E-EL-H1289), GPX3 (Reed Biotech Ltd, ml057772), ATRN (Reed Biotech Ltd, RE2610H), and FGB (Reed Biotech Ltd, RE2242H) were measured using commercial ELISA kits, with all procedures strictly performed according to the manufacturers’ instructions.

### Protein preprocessing

After centrifugation at 12,000g for 10 minutes at 4°C to remove debris, the supernatant was processed using the Thermo Scientific Pierce™ Top 14 Abundant Protein Depletion Column. The protein concentration was determined using the BCA method. All procedures were conducted following standardized protocols to minimize batch variation.

### Trypsin digestion

The protein solution was treated with 5 mM dithiothreitol (DTT) at 56°C for 30 minutes to perform the reduction reaction. Subsequently, an alkylation reaction was carried out at room temperature using 11 mM iodoacetamide (IAM) in a dark environment for 15 minutes. After the alkylation was completed, the sample was transferred to an ultrafiltration tube, and the filter membrane-assisted sample preparation (FASP) method was employed for proteolysis. First, the sample was subjected to three exchanges with 8 M urea solution at room temperature under a centrifugal force of 12,000 g for 20 minutes each to remove impurities. Then, the sample was further washed with 200 mM triethylammonium bicarbonate (TEAB) through three exchanges. Trypsin was added at a ratio of 1:50 (trypsin to protein mass) and allowed to digest overnight at room temperature. After digestion, the mixture was centrifuged at 12,000 g for 10 minutes at room temperature to collect the peptides, and this operation was repeated twice to ensure complete recovery. Finally, the collected peptides were desalted using Strata X solid-phase extraction columns to remove impurities and purify the sample.

### Liquid chromatography-mass spectrometry analysis

The peptide segments were separated using the Vanquish Neo ultra-high-performance liquid chromatography system. The mobile phase A (0.1% formic acid + 2% acetonitrile) and B (0.1% formic acid + 90% acetonitrile) were eluted in a gradient: from 0 to 16 minutes (7% to 20% B), from 16 to 24 minutes (20% to 32% B), from 24 to 27 minutes (32% to 80% B), and from 27 to 30 minutes (maintaining 80% B) at a flow rate of 500 nl/min. Mass spectrometry parameters included an ion source voltage of 2300V, full scan resolution of 30000 (m/z 390–810), MS/MS scan resolution of 30000, and HCD fragmentation energy of 25%/30%/35%. Data collection was performed in DDA mode to meet the data analysis requirements of small-scale experiments, using Spectronaut 17.0 software and the Pulsar search engine to search the human protein database (hom_sapiens_9606_sp_20230103, 20389 entries) and the reverse decoy database. Parameter settings included a maximum missed cleavage of 2, fixed modifications (carbamidomethylation of cysteine), variable modifications (N-terminal acetylation, oxidation of methionine), with FDR < 1% at the PSM, peptide, and protein levels.

### Bioinformatics analysis and ELISA data analysis

The subcellular localization of the identified proteins was predicted using the WOLF PSORT tool (available at WOLF PSORT), with default settings applied. For functional annotation, the identified proteins were annotated with Gene Ontology (GO) terms using the UniProt-Gene Ontology Annotation (UniProt-GOA) database hosted by the European Bioinformatics Institute (UniProt-GOA). Protein IDs were first converted to UniProt IDs and subsequently mapped to corresponding GO IDs. GO enrichment analysis was performed using a two-tailed Fisher’s exact test, with a significance threshold set at *P* < 0.05. Additionally, the identified proteins were annotated through the KEGG database (KEGG), and enriched pathways were mapped based on the annotation information. Dynamic clustering analysis was performed using the Mfuzz algorithm. Protein expression values were Log2-transformed, and proteins with a standard deviation (SD) > 0.5 were selected for analysis. The cluster number was set to k = 6, and the fuzzifier parameter was set to m = 2. Plasma protein concentrations measured by ELISA were analyzed using GraphPad Prism 10.0. Differences between groups were assessed using the Mann-Whitney U test, and *P* < 0.05 was considered statistically significant.

## Results

### Overall research design and comprehensive analysis of plasma proteomics

**Fig 1:**
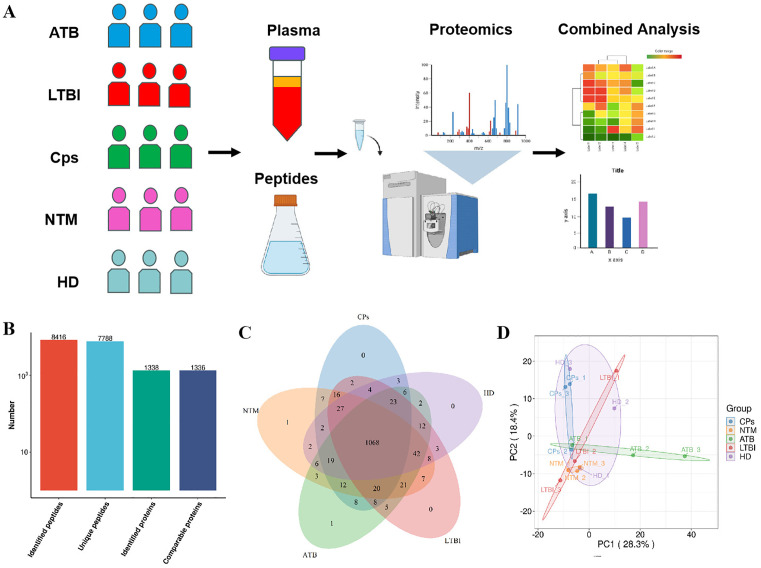
Quantitative proteomic analysis of human plasma samples. **(A)** Overview of the study design and experimental workflow. **(B)** The overall profile of identified peptides and proteins. **(C)** The number of identified peptides and proteins. **(D)** Principal component analysis (PCA) scatter plots were conducted on all identified features to exploramine the largestprimary sources of variance within each omics dataset.

This study established a multidimensional analytical framework based on plasma proteomics, as illustrated in Fig 1A. The aim is to develop a molecular identification system for ATB, LTBI, and NTM, while also uncovering prognostic biomarkers related to ATB. Through the analysis of plasma protein expression profiles from five groups: ATB, LTBI, NTM, CPs, and HD, we identified a total of 1,338 non-redundant proteins (Fig 1B, S1 Table), of which 1,068 proteins were enriched across all five groups (Fig 1C). The multidimensional quality control system validated the reliability of the data: Pearson correlation coefficients and relative standard deviations indicated excellent experimental reproducibility (S1 Fig A,B). The quality control system encompasses a range of multidimensional evaluation metrics, including peptide length distribution (S1 Fig C), peptide quantity distribution (S1 Fig D), protein identification coverage (S1 Fig E), and molecular weight distribution characteristics (S1 Fig F), systematically assessing the stability of sample preparation and mass spectrometry detection. The observed peptide length was predominantly distributed between 7 and 20 amino acids, which falls within the expected optimal range for tryptic digestion and mass spectrometry fragmentation, confirming proper sample preparation. Furthermore, the molecular weight of identified proteins was evenly distributed across a wide range, indicating no significant loss of proteins of specific molecular weights during experimental processing. All raw data and annotation information are included in S1 Table and S2 Table. Principal Component Analysis (PCA) revealed that Principal Component 1 (PC1) and Principal Component 2 (PC2) accounted for 28.3% and 18.4% of the total variance, respectively, significantly distinguishing the molecular characteristics of different clinical groups (Fig 1D). This indicates a significant difference in plasma proteomics among various diseases.

### Analysis of plasma protein expression profiles specific to ATB, LTBI, and NTM populations

**Fig 2:**
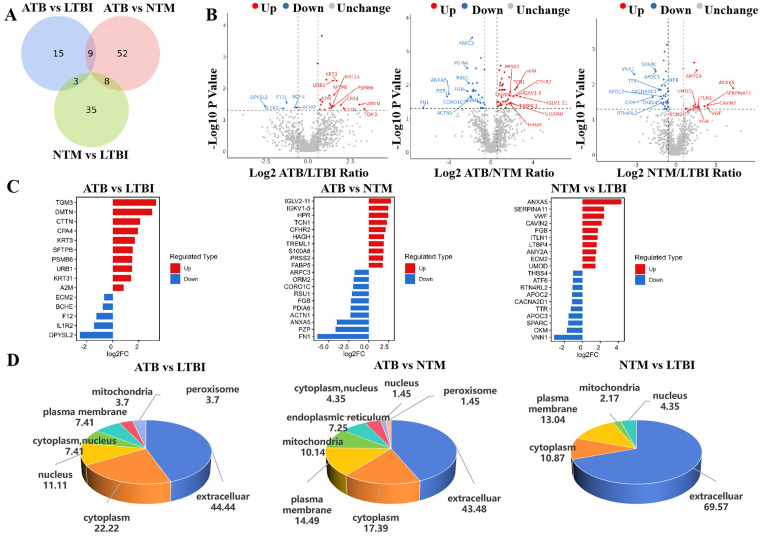
Comparative analysis of plasma proteins among the ATB, LTBI, and NTM groups. **(A)** The Venn diagram illustrates the specific and shared differential proteins among the comparisons of the groups. **(B)** The volcano plot presents the distribution characteristics of differentially expressed proteins in each group. **(C)** The heatmap displays the top 10 most significantly upregulated and downregulated proteins in the comparisons among the groups. **(D)** Analysis of the subcellular localization characteristics of differentially expressed proteins.

To elucidate the differences in plasma proteomes between tuberculosis infections and non-tuberculous infections, we conducted pairwise comparisons among samples from ATB, LTBI, and NTM groups, The unique and overlapping DEPs identified from these three pairwise comparisons are summarized in the Venn diagram (Fig 2A), showing a total of 142 DEPs across all comparisons (including overlapping proteins). Specifically, the analysis revealed 27 DEPs (22 upregulated and 5 downregulated) in the ATB-LTBI group, 69 DEPs (41 upregulated and 28 downregulated) in the ATB-NTM group, and 46 DEPs (13 upregulated and 33 downregulated) in the NTM-LTBI group (Fig 2A,2B). Notably, the ATB-NTM and NTM-LTBI groups share 8 core DEPs (S3 Table), suggesting their potential utility in the differential diagnosis of NTM. Analysis of the top 10 differential proteins from the pairwise comparisons (Fig 2C) reveals that the ATB-related DEPs in the comparison with LTBI primarily involve molecules related to inflammation/immune response (such as the downregulation of acute phase reactant protein A2M, complement component F12, and IL-1 receptor IL-1R2) as well as epithelial barrier function proteins (TGM3, KRT3/KRT31). In the comparison between ATB and NTM, the ATB group exhibited a specific upregulation of immunoglobulin light chains (IGLV2–11, IGKV1–5) and innate immune effector molecules (S100A8), while the NTM group showed significant downregulation of cytoskeletal regulatory proteins (ARPC3, ACTN1) and fibronectin (FN1). Notably, the differentially expressed proteins in the NTM compared to the LTBI group were significantly enriched in lipid metabolism-related molecules (apolipoprotein APOC3, thyroxine transport protein TTR), vascular regulatory proteins (VWF, THBS4), and extracellular matrix components (ECM2, SPARC), with APOC3 showing significant downregulation, a finding that aligns closely with the lipid metabolic disorder characteristics unique to NTM infection.

Subcellular localization analysis revealed that the DEPs in the ATB, LTBI, and NTM groups exhibited significant compartmentalization (Fig 2D). The differential proteins between the ATB and LTBI groups were primarily distributed in the extracellular domain (44.44%), cytoplasm (22.22%), and nucleus (11.11%); the differential proteins between the ATB and NTM groups were mainly found in the extracellular domain (43.48%), cytoplasm (17.39%), cell membrane (14.49%), and mitochondria (10.14%); while the differential proteins between the NTM and LTBI groups were predominantly located in the extracellular domain (69.57%), followed by cytoplasm (13.04%), nucleus (4.35%), and mitochondria (2.17%).

### Comparative analysis of protein functional characteristics among ATB, LTBI, and NTM populations

**Fig 3:**
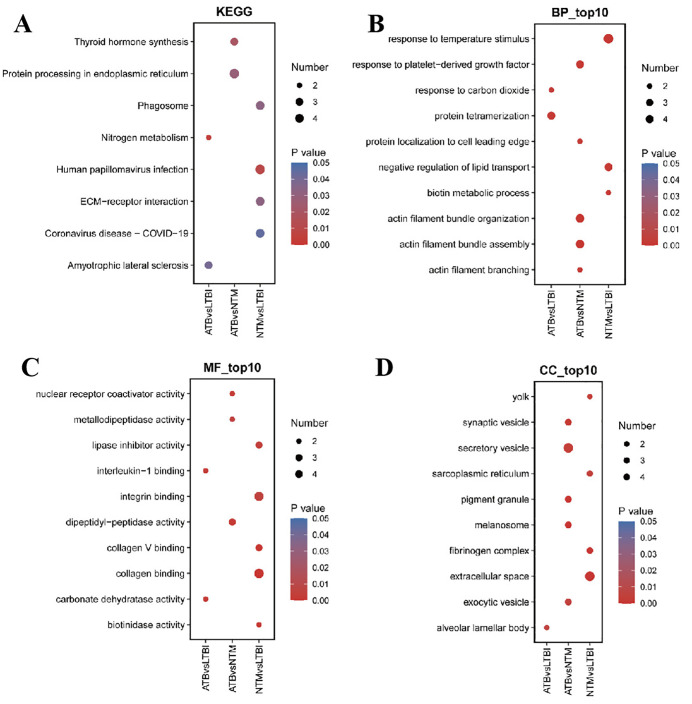
Functional characteristic comparison of plasma proteins among the ATB, LTBI, and NTM groups. **(A)** KEGG pathway analysis reveals the metabolic pathway characteristics of differentially expressed proteins in each group. **(B)** Biological process (BP) functional annotation analysis. **(C)** Distribution characteristics of cellular components (CC). **(D)** Molecular function (MF) classification analysis.

Systematic interpretation based on KEGG and Gene Ontology (GO) pathway analyses revealed that the functional features of differentially expressed proteins in the ATB, LTBI, and NTM groups exhibited significant disease-state specificity (Fig 3). KEGG pathway analysis further validated these findings (Fig 3A): The ATB-LTBI group showed significant enrichment in nitrogen metabolism (immune cell metabolic reprogramming) and amyotrophic lateral sclerosis (neurodegenerative pathways). The ATB-NTM group was specifically associated with thyroid hormone synthesis (endocrine-immune crosstalk regulation) and endoplasmic reticulum protein processing (stress-induced protein quality control). The NTM-LTBI group displayed high expression in pathways related to phagosomes (macrophage dysfunction), coronavirus infection (viral cooperative mechanisms), and extracellular matrix interaction (tissue fibrosis).

Based on Gene Ontology (GO) analysis, at the Biological Process (BP) level (Fig 3B), the differentially expressed proteins in the ATB-LTBI group are primarily involved in protein localization to the cell leading edge and response to carbon dioxide. The ATB-NTM group is significantly enriched in response to platelet-derived growth factor and actin filament bundle assembly/organization, as well as actin filament branching. In contrast, the NTM-LTBI group primarily relates to response to temperature stimulus, negative regulation of lipid transport, and biotin metabolic process.At the CC level (Fig 3C), the differential proteins are primarily localized in secretory vesicles, synaptic vesicles, exocytic vesicles, extracellular space, specialized organelles (such as yolk, sarcoplasmic reticulum, pigment granules, melanosomes, and alveolar lamellar bodies), as well as in the fibrinogen complex. At the MF level (Fig 3D), the differential proteins in the ATB-LTBI group are significantly enriched in interleukin-1 binding and carbonate dehydratase activity; in contrast, the ATB-NTM group predominantly exhibits dipeptidyl-peptidase activity and nuclear receptor coactivator activity; The NTM-LTBI group is characterized by integrin binding, collagen binding, collagen V binding, and lipase inhibitor activity.

The protein interaction network analysis based on the STRING database reveals that the network of the ATB-LTBI group centers around GPX3, forming an oxidative stress clearance module with CAT and BCHE. CA3 interacts with CA2 to regulate the pH homeostasis of the microenvironment, while TGM3 interacts with KRT2 (S2 Fig A). The network of the ATB-NTM group consists of FN1, ACTN1, and CLIC4, forming a core module for actin remodeling, which cooperates with LTBP1 to regulate cellular mechanics, and an interaction between C4A and SERPINA1 is observed (S2 Fig B). In contrast, the NTM-LTBI group network is centered on ITLN1, which connects with APOC2, APOC3, FGB, and ADAMTS, while interactions between PROC and F13B are also present (S2 Fig C). These interaction networks reveal specific molecular characteristics under different infection statuses.

### Protein expression changes related to the therapeutic effect of tuberculosis treatment

**Fig 4:**
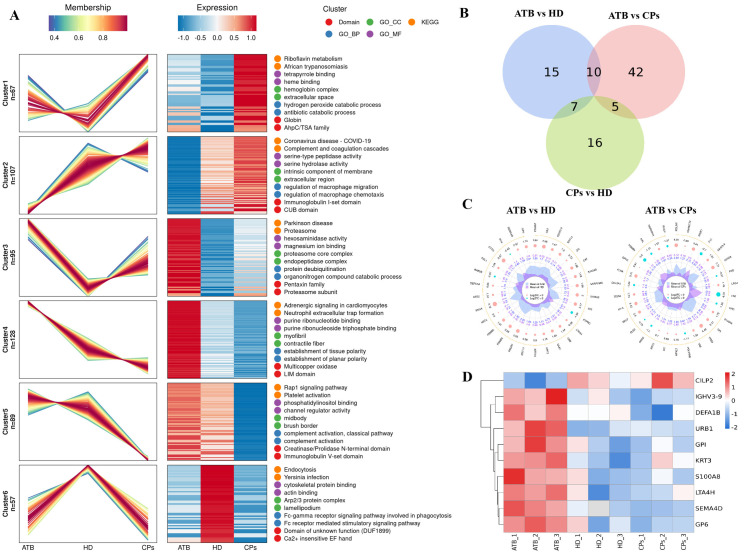
Comparative analysis of plasma proteins among the ATB, HD, and CPs groups. **(A)** Expression patterns and functional analysis of proteins in the ATB, HD, and CPs cohorts. **(B)** The Venn diagram shows the specific and shared differential proteins among the comparisons of the groups. **(C)** The bar plot presents differentially expressed proteins in the comparisons between ATB and HD, and ATB and CPs. **(D)** The heatmap illustrates the expression patterns of the common differentially expressed proteins shown in **(C)**.

Based on Mfuzz dynamic clustering analysis, this study reveals significantly distinct protein dynamic reprogramming patterns among ATB, HD, and CPs, identifying six treatment response-related modules (Fig 4A). Cluster 1 (n = 67) is expressed at low levels in the ATB/HD group but upregulated post-treatment, primarily involved in riboflavin metabolism, tetrapyrrole binding, and heme binding functions. Cluster 2 (n = 107) shows increased expression in the CPs group, associated with complement and coagulation cascades, serine hydrolase activity, and macrophage chemotaxis regulation. Cluster 3 (n = 95) is highly expressed in the ATB group and partially reversed post-treatment, related to proteasome core complex assembly, parkinson disease pathway, magnesium ion binding, and pentaxin family protein-mediated ubiquitin-proteasome system. Cluster 4 (n = 128) was significantly downregulated after treatment, participating in adrenergic signaling in adrenergic signaling in cardiomyocytes, purine ribonucleoside triphosphate binding, and contractile fiber structure. Cluster 5 (n = 89) sharply decreased after treatment, involving platelet activation, phosphatidylinositol binding, and complement activation, classical pathway. Cluster 6 (n = 57) exhibited dynamic fluctuation in expression, primarily participating in endocytosis, cytoskeletal protein binding, and yersinia infection response.

The Venn diagram of differential proteins (Fig 4B) shows that the number of specific differential proteins between the ATB and CPs groups is the highest (42), significantly exceeding that between the CPs and HD groups (10) and between the ATB and HD groups (15). The radar chart (Fig 4C) further elucidates the dynamic of differential proteins between ATB and HD, as well as between ATB and CPs. The heatmap (Fig 4D) confirms the expression heterogeneity of the 10 common differential proteins (CILP2, IGHV3–9, DEFA1B, URB1, GPI, KRT3, S100A8, LTA4H, SEMA4D, GP6) between the ATB and HD groups and between the ATB and CPs groups.

### Plasma biomarker expression levels and diagnostic efficacy analysis

**Fig 5:**
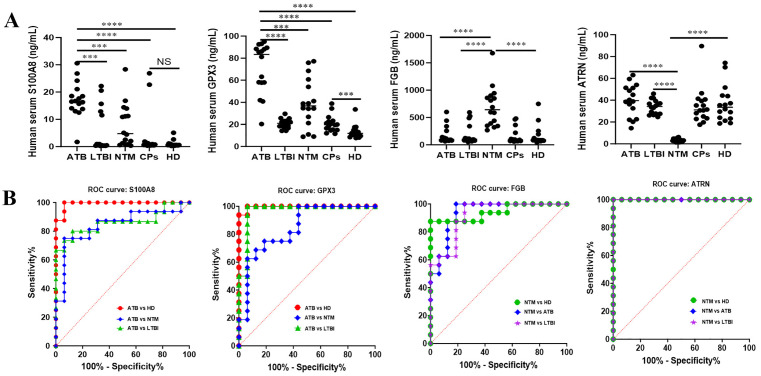
Analysis of expression levels and diagnostic efficacy of plasma S100A8, GPX3, FGB, and ARTN. **(A)** Concentrations of S100A8, GPX3, FGB, and ARTN in Plasma samples from individuals with ATB, LTBI, NTM, CPs, and HD, as determined by enzyme-linked immunosorbent assay. **(B)** Receiver operating characteristic (ROC) curves demonstrating the diagnostic performance of each biomarker across different disease groups.

This study systematically analyzed the expression characteristics of four Plasma biomarkers in ATB, LTBI, NTM, CPs, and HD individuals by enzyme-linked immunosorbent assay (Fig 5A). The results showed that compared with the LTBI, NTM, and HD groups, the Plasma concentrations of S100A8 and GPX3 in the ATB group were significantly increased. It is particularly noteworthy that different recovery patterns were observed in cured tuberculosis patients (CPs): S100A8 levels returned to normal levels showing no statistical difference from the HD group, while GPX3 levels, although significantly reduced compared to the ATB group, remained maintained at levels higher than the HD group (*P* < 0.05). In addition, in the NTM group, the Plasma concentration of FGB was significantly higher than that in the ATB, LTBI, and HD groups, while the ATRN concentration was significantly lower than that in the ATB, LTBI, and HD groups.

Diagnostic efficacy was evaluated by receiver operating characteristic curve analysis (Fig 5B, Table 1). In distinguishing ATB from HD, both S100A8 (AUC = 0.992) and GPX3 (AUC = 0.996) exhibited excellent diagnostic performance, with their sensitivity and specificity reaching 100%/93.8% and 93.8%/100%, respectively. In discriminating ATB from LTBI, GPX3 (AUC = 0.969) showed high discriminatory ability (sensitivity 100%, specificity 93.8%), while S100A8 (AUC = 0.863) followed (sensitivity 80.0%, specificity 87.5%). Both also showed certain discriminatory ability in distinguishing ATB from NTM (AUCs were 0.844 and 0.848, respectively). When distinguishing NTM from HD, the AUC of ATRN reached 1.000 (sensitivity 100%, specificity 100%), and the AUC of FGB was 0.941 (sensitivity 87.5%, specificity 100%). Similarly, when distinguishing ATB from NTM, ATRN also exhibited good discriminatory ability (AUC = 1.000, sensitivity 100%, specificity 100%), and FGB also showed good diagnostic efficacy (AUC = 0.938, sensitivity 100%, specificity 81.3%).

**Table 1:** Diagnostic performance of S100A8, GPX3, FGB, and ATRN.

Protein name	Groups	Sensitivity	Specificity	AUC
S100A8	ATB vs HD	100%	93.8%	0.992
	ATB vs NTM	75.0%	93.8%	0.844
	ATB vs LTBI	80.0%	87.5%	0.863
GPX3	ATB vs HD	93.8%	100%	0.996
	ATB vs NTM	68.8%	87.5%	0.848
	ATB vs LTBI	100%	93.8%	0.969
FGB	NTM vs HD	87.5%	100%	0.941
	NTM vs ATB	100%	81.3%	0.938
	NTM vs LTBI	100%	75.0%	0.918
ATRN	NTM vs HD	100%	100%	1.00
	NTM vs ATB	100%	100%	1.00
	NTM vs LTBI	100%	100%	1.00

ATB, active tuberculosis; LTBI, latent tuberculosis infection; NTM, nontuberculous mycobacteria; CPs, cured patients; HD, healthy donors; AUC, area under the curve.

AUC values were classified as follows: > 0.9 were considered “excellent”; > 0.8 to ≤0.9, “accurate”; > 0.7 to ≤0.8, “moderately accurate”; and 0.5 to ≤0.7, “uninformative”.

## Discussion

This study systematically reveals the molecular characteristics and therapeutic dynamic response mechanisms at different stages of mycobacterial infection by integrating plasma proteomics with multidimensional bioinformatics analysis. Compared to previous studies, this research has achieved significant breakthroughs across multiple dimensions. Firstly, in terms of differential diagnosis, the identification of 132 disease-specific DEPs provides a new molecular framework for the precise classification of ATB/LTBI/NTM. Traditional diagnostic techniques such as the IGRA can detect tuberculosis infections but fail to distinguish between active and latent infection states [13]. Furthermore, molecular detection methods like Xpert MTB/RIF have limited capability in differentiating NTM [14]. This study identifies 69 DEPs in the ATB-NTM group, such as FN1 and HPR, which not only provide specific biomarkers for distinguishing between tuberculosis and non-tuberculous mycobacterial infections but also highlight the significance of mitochondrial oxidative stress and extracellular matrix damage in the pathogenesis of NTM. To validate the reliability of the proteomics results, we performed experimental verification of FGB and ATRN using ELISA. The results showed that plasma FGB levels in the NTM group were significantly higher than those in the HD, ATB, and LTBI groups, which is consistent with the proteomics data. As the beta subunit of fibrinogen, FGB is an important acute-phase reaction protein that is widely involved in the regulation of inflammation and tissue repair processes [15]. Although its role in NTM infection remains unclear, studies have shown that FGB is dysregulated in various diseases, such as renal cell carcinoma, malignant pulmonary nodules, and breast cancer, and is associated with disease progression [16–18]. The significant increase in plasma FGB levels observed in the NTM-infected population in this study suggests that FGB may be involved in NTM-related pulmonary pathological processes, and its mechanisms and clinical significance warrant further investigation. In contrast, ATRN levels were significantly decreased in the NTM group. ATRN is a member of the cell adhesion and guidance protein family that contains CUB domains, EGF-like domains, and kelch repeats [19]. It has been demonstrated to be involved in immune regulation and chemotactic processes, and can modulate T-cell activation and monocyte mobilization [20], but its role in mycobacterial infections has not yet been clarified. The significant downregulation of ATRN observed in NTM patients in this study suggests that immunomodulatory pathways may be impaired, which might be associated with the aberrant immune responses characteristic of NTM infection. Additionally, the study identifies 27 DEPs in the ATB-LTBI group, including F12, ECM2, and A2M, which, in conjunction with the research by Chend et al. [21] on acute phase proteins, reveal critical mechanisms underlying the immune metabolic regulation in tuberculosis. Our results not only confirm the important role of A2M in active tuberculosis but also uncover a regulatory network involving A2M, F12, and ECM2. Meanwhile, Chend et al. demonstrated that A2M combined with CRP and SAP can accurately predict treatment failure. These findings indicate that acute phase proteins are not only key participants in the immune metabolic imbalance of tuberculosis but also effective predictors of treatment response, providing new insights for the precise diagnosis and treatment of tuberculosis.

This study systematically reveals significant differences in molecular functions among the ATB, LTBI, and NTM groups through KEGG and GO pathway analyses. The notable enrichment of the nitrogen metabolism pathway in the ATB-LTBI group is particularly striking. This finding is highly consistent with the results of Zimmermann et al. [22], who confirmed through integrated metabolomics and transcriptomics analyses that MT can actively regulate the host cell’s nitrogen metabolism pathway, especially by reprogramming arginine metabolism to promote its own survival and immune evasion. More specifically, our data indicate abnormal expression of key enzymes involved in the urea cycle and glutamine metabolism, which may directly affect the antimicrobial functions of macrophages, creating a favorable intracellular environment for *Mtb*. The significant enrichment of the endoplasmic reticulum protein processing pathway in the ATB-NTM group may be related to endoplasmic reticulum stress-mediated apoptosis. Meanwhile, the aberration in the phagosome pathway observed in the NTM-LTBI group may indicate how NTM suppresses the bactericidal function of macrophages by disrupting lysosomal maturation. This finding aligns with the classic study by Sturgill-Koszycki et al. [23], which revealed an immune evasion mechanism whereby NTM prevents phagosome acidification by excluding the vesicular proton-ATPase. Protein-protein interaction network analysis identified key regulatory nodes such as GPX3, FN1 and ITLN1. In the ATB-LTBI group, GPX3, as the core molecule of oxidative stress response, together with CAT and BCHE, constitutes an antioxidant defense network. Consistently, our ELISA validation results confirmed that the plasma GPX3 level specifically increased during active tuberculosis and gradually returned to the baseline level after cure. As the main extracellular antioxidant enzyme responsible for eliminating peroxides and maintaining REDOX balance [24], the upregulation of GPX3 is likely to represent a host compensatory mechanism to combat infection-induced oxidative stress. This finding is consistent with the protective effects of GPX3 reported in other pathological conditions, such as its ability to inhibit vascular inflammation and oxidative stress demonstrated by Hauffe et al. [25], thereby emphasizing the core role of REDOX imbalance in the pathogenesis of tuberculosis. In the ATB-NTM group, the cytoskeletal remodeling module composed of FN1 and ACTN1 is highly consistent with the mechanisms of cellular mechanical changes induced by mycobacterial infection reported by Bryant et al. [26].

The analysis of dynamic therapeutic responses reveals the temporal reprogramming patterns of host protein networks during tuberculosis treatment. The discovery of six therapeutic response modules systematically elucidates the molecular transformation process from inflammatory activation to tissue repair. For instance, the activation of Cluster 1/2 (riboflavin metabolism and complement coagulation pathway) post-treatment may promote tissue repair by regulating redox balance and coagulation homeostasis, a phenomenon corroborated by Roca et al. [27] regarding the enhancement of host antibacterial immunity through riboflavin metabolism. The therapeutic response modules identified in this study (such as the partial reversal of proteasome and myocardial signaling pathways in Cluster 3/4) may provide new insights into monitoring organ toxicity induced by anti-tuberculosis drugs. Furthermore, the delayed regression of Cluster 5/6 (platelet activation and cytoskeleton pathway) suggests that microvascular damage and cytoskeletal remodeling may persist after tuberculosis treatment, aligning with the clinical observations of Walzl et al. [28] regarding residual pulmonary fibrosis post-tuberculosis treatment. This study confirmed the high specificity of S100A8 expression in patients with ATB through ELISA, as compared to HD, LTBI, and NTM. After treatment, S100A8 levels returned to healthy levels. This finding is corroborated by several studies: Sitoe et al. [29] demonstrated that plasma S100A8 levels significantly decreased during the course of tuberculosis treatment, Jiang et al. [30] confirmed that S100A8 is the best biomarker for distinguishing between active and latent tuberculosis, while Muefong et al. [31] further established that S100A8 levels are significantly correlated with neutrophil counts and the degree of lung damage. Additionally, Ren et al. [32] not only validated the diagnostic value of S100A8 for spinal tuberculosis but also found a strong positive correlation with inflammatory markers (CRP, ESR). Collectively, these results indicate that S100A8 can serve as a specific diagnostic marker for active tuberculosis, and its dynamic changes can effectively reflect disease activity and treatment response, providing important molecular monitoring indicators for the clinical management of tuberculosis.

This study confirms the expression heterogeneity of 10 differential proteins shared between the ATB and HD groups, as well as between the ATB and CPs groups, through proteomics analysis. These findings are significantly related to previous tuberculosis research. The expression characteristics of LTA4H are consistent with the results of Tobin et al. [33], who demonstrated through zebrafish models and human genetics studies that LTA4H influences tuberculosis susceptibility by regulating the balance of leukotriene B4 (LTB4) and lipoxin. This study revealed that plasma URB1 levels were significantly elevated in patients with active tuberculosis and markedly decreased after treatment, suggesting a potential association between URB1 and disease activity in tuberculosis. URB1 is a key regulator of ribosomal RNA processing and nucleolar function, playing an essential role in maintaining cell proliferation [34]. During *Mtb* infection, the upregulation of URB1 may reflect nucleolar hyperactivity accompanying immune cell (e.g., lymphocyte) activation, which supports their rapid proliferation and protein synthesis demands. The expression pattern of DEFA1B corroborates the findings of Wang et al. [35] regarding the potential role of defensins in HIV/TB co-infection, where bioinformatics analysis identified specific expression characteristics of defensin family genes, including DEFA1B, in tuberculosis. Together, these molecules constitute a tuberculosis-specific inflammatory response network, providing an important theoretical basis for understanding the molecular characteristics of tuberculosis and developing novel diagnostic markers.

Despite the significant advancements made in this study, certain limitations remain. Firstly, the sample size of the research cohort is relatively limited, and future studies should validate the universality of the biomarkers through multi-center large-sample cohorts. Secondly, the biological functional validation of the proteomic data requires further investigation, such as exploring the regulatory mechanisms of key node molecules (e.g., GPX3 or ITLN1) in infection models using gene editing technologies. Additionally, the NTM group was not further subdivided into different species, which may affect the species-specific analysis of the biomarkers; this area needs to be explored in conjunction with NTM subtype classification in subsequent research.

In summary, this study systematically analyzed the molecular characteristic spectrum and therapeutic dynamics of mycobacterial infections using high-resolution proteomics technology, providing important theoretical foundations and translational tools for the precise diagnosis, individualized treatment, and prognostic assessment of tuberculosis and NTM. Future research could integrate multi-omics data (such as transcriptomics and metabolomics) to further reveal the comprehensive network regulatory mechanisms of host-pathogen interactions, thereby promoting the development of precision medicine in tuberculosis.

## Conclusions

This study systematically reveals the molecular characteristics and therapeutic dynamic response mechanisms at different stages of mycobacterial infection by integrating plasma proteomics with multidimensional bioinformatics analysis. The ATB/LTBI/NTM differential diagnosis system, constructed based on 132 disease-specific differential proteins, overcomes the technical bottlenecks of traditional methods in distinguishing infection states. The dynamic analysis of six temporal therapeutic response modules elucidates the reprogramming patterns of the host protein network in tuberculosis treatment. The validation of the key biomarker S100A8 confirms its high reliability as a kinetic indicator of active tuberculosis inflammation, providing sensitive molecular tools for clinical efficacy monitoring. This research not only establishes a precise molecular typing framework for mycobacterial infections but also lays a multidimensional theoretical foundation for the formulation of individualized treatment strategies and prognostic assessments, promoting the paradigm shift of tuberculosis diagnosis from empirical medicine to precision medicine.

## Supporting information

S1 Fig Data quality control analysis. (A-B) The Pearson correlation coefficient (PCC) and relative standard deviation (RSD) demonstrate good reproducibility across the entire proteomic samples. (C) Distribution characteristics of peptide lengths. (D) Distribution of the number of peptides. (E) Coverage of identified proteins. (F) Distribution characteristics of protein molecular weights.
(TIF)

S2 Fig Differentially expressed protein interaction network analysis. (A) Differential protein interaction network for the ATB vs LTBI group. (B) Differential protein interaction network for the ATB vs NTM group. (C) Differential protein interaction network for the NTM vs LTBI group.
(TIF)

S1 Table Descriptive characteristics of the study participants. (XLSX)

S2 Table Summary of MS/MS spectrum database search analysis: identified peptides, unique peptides, and proteins. (XLSX)

S3 Table Differential expression analysis of proteins across comparative groups: fold change and regulation patterns. (XLSX)

S4 Table Shared differentially expressed proteins (DEPs) Between ATB-NTM and NTM-LTBI Groups. (XLSX)
