# Supplementary figures and images for: Identification of diagnostic and prognostic biomarkers for tuberculosis based on plasma proteomics
Source: PLoS One. 2025 Dec 22;20(12):e0339558. doi: 10.1371/journal.pone.0339558 (PMC12721542; doi:10.1371/journal.pone.0339558)

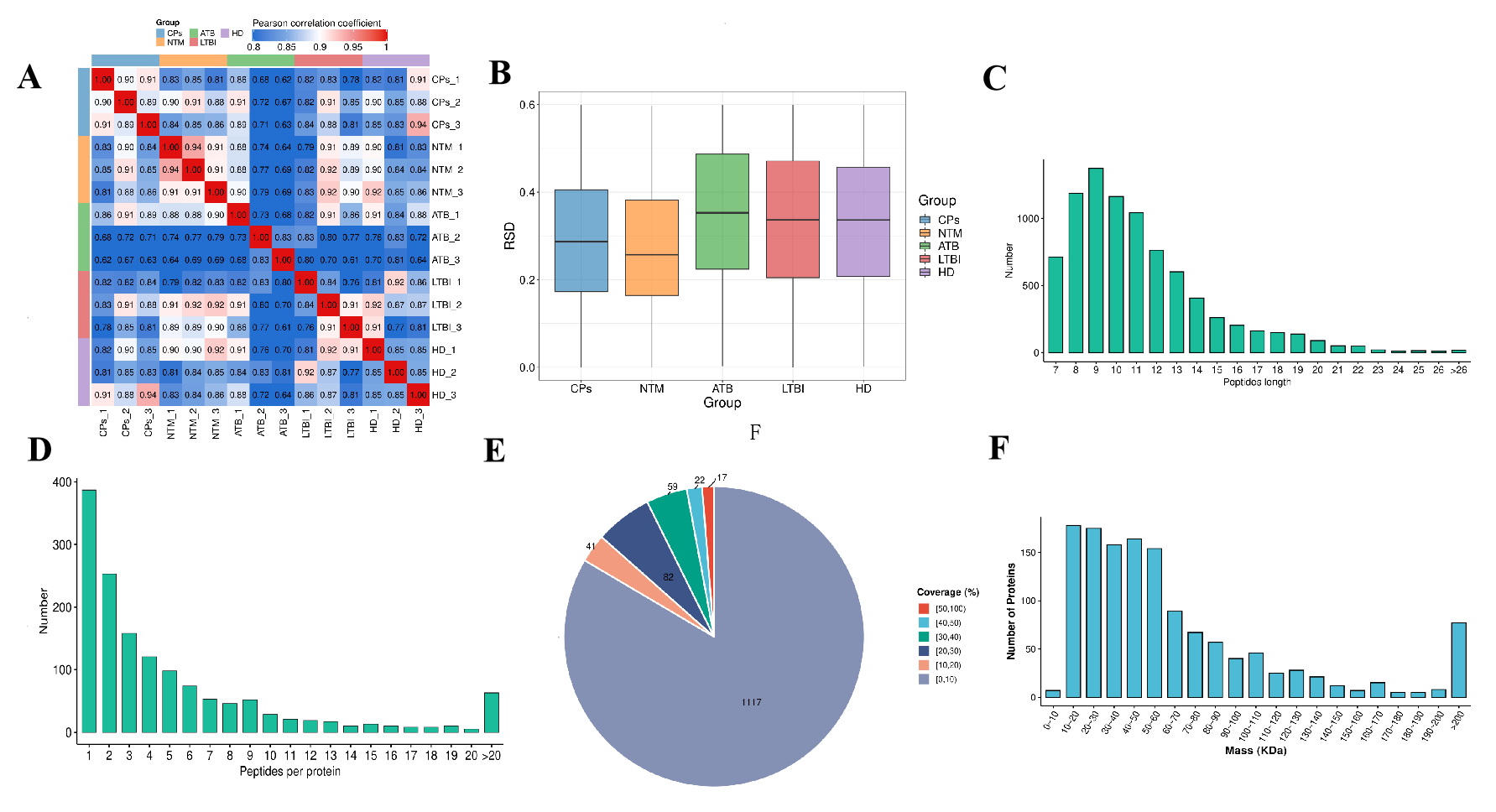

Supplement: S1 Fig — (A-B) The Pearson correlation coefficient (PCC) and relative standard deviation (RSD) demonstrate good reproducibility across the entire proteomic samples. (C) Distribution characteristics of peptide lengths. (D) Distribution of the number of peptides. (E) Coverage of identified proteins. (F) Distribution characteristics of protein molecular weights. (TIF) [file pone.0339558.s001.tif]

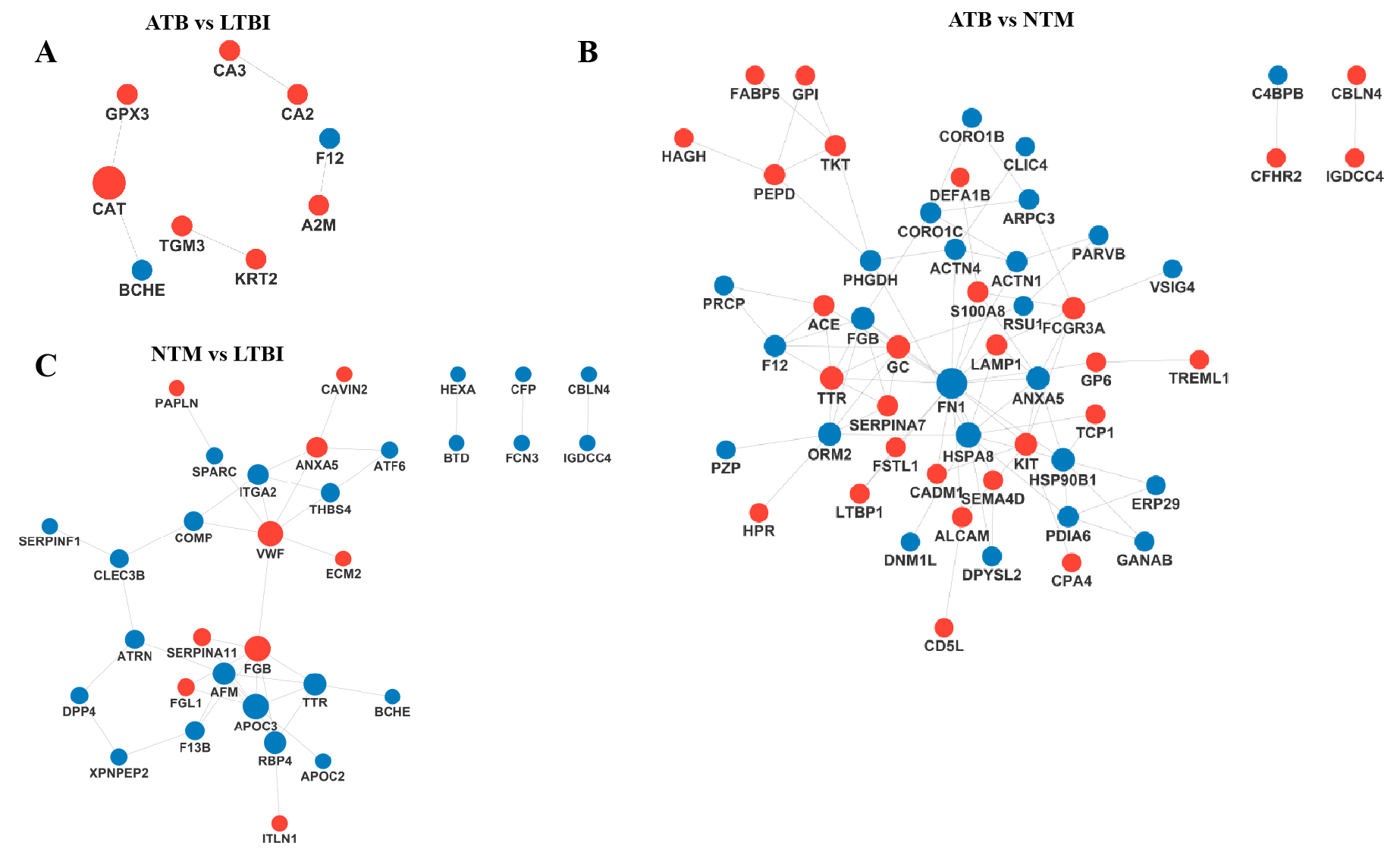

Supplement: S2 Fig — (A) Differential protein interaction network for the ATB vs LTBI group. (B) Differential protein interaction network for the ATB vs NTM group. (C) Differential protein interaction network for the NTM vs LTBI group. (TIF) [file pone.0339558.s002.tif]
